# Publisher Correction: Comparative validation of AI and non-AI methods in MRI volumetry to diagnose Parkinsonian syndromes

**DOI:** 10.1038/s41598-023-33774-z

**Published:** 2023-05-03

**Authors:** Joomee Song, Juyoung Hahm, Jisoo Lee, Chae Yeon Lim, Myung Jin Chung, Jinyoung Youn, Jin Whan Cho, Jong Hyeon Ahn, Kyungsu Kim

**Affiliations:** 1 Department of Neurology and Neuroscience Center, Samsung Medical Center, Sungkyunkwan University School of Medicine, Seoul, Republic of Korea; 2 Medical AI Research Center, Research Institute for Future Medicine, Samsung Medical Center, Seoul, Republic of Korea; 3 Department of Biostatistics, Columbia University, New York, NY USA; 4 Department of Electrical and Computer Engineering, University of Maryland, College Park, MD USA; 5 Department of Medical Device Management and Research, SAIHST, Sungkyunkwan University, Seoul, Republic of Korea; 6 Department of Radiology, Samsung Medical Center, Sungkyunkwan University School of Medicine, Seoul, Republic of Korea; 7 Department of Data Convergence and Future Medicine, Sungkyunkwan University School of Medicine, Seoul, Republic of Korea; 8 Department of Radiology, Massachusetts General Brigham and Harvard Medical School, Boston, MA USA

Correction to: *Scientific Reports* 10.1038/s41598-023-30381-w, published online 01 March 2023

The original online version of this Article was revised: In the original version of this Article, the Supplementary File, which was included with the initial submission, was omitted from the Supplementary Information section. The correct Supplementary Information file now accompanies the original Article.

