# Supplementary material for: Comparative validation of AI and non-AI methods in MRI volumetry to diagnose Parkinsonian syndromes
Source: Sci Rep. 2023 Mar 1;13:3439. doi: 10.1038/s41598-023-30381-w (PMC10156821; doi:10.1038/s41598-023-30381-w)
Supplement: Supplementary file 1 — Supplementary Information. [file 41598_2023_30381_MOESM1_ESM.pdf]

Comparative Validation of AI and non-AI Methods in MRI Volumetry to Diagnose Parkinsonian Syndromes

Joomee Song[1,+], Juyoung Hahm[2,3,+], Jisoo Lee[2,4], Chae Yeon Lim[2,5], Myung Jin Chung[2,6,7], Jinyoung Youn[1], Jin Whan Cho[1], Jong Hyeon Ahn[1,*], and Kyungsu Kim[2,7,8,*]

[1]Department of Neurology and Neuroscience Center, Samsung Medical Center, Sungkyunkwan University School of Medicine, Seoul, Republic of Korea
[2]Medical AI Research Center, Research Institute for Future Medicine, Samsung Medical Center, Seoul, Republic of Korea
[3]Department of Biostatistics, Columbia University, New York, NY, USA
[4]Department of Electrical and Computer Engineering, University of Maryland, College Park, MD, USA
[5]Department of Medical Device Management and Research, SAIHST, Sungkyunkwan University, Seoul, Republic of Korea
[6]Department of Radiology, Samsung Medical Center, Sungkyunkwan University School of Medicine, Seoul, Republic of Korea
[7]Department of Data Convergence and Future Medicine, Sungkyunkwan University School of Medicine, Seoul, Republic of Korea
[8]Department of Radiology, Massachusetts General Brigham and Harvard Medical School, Boston, MA, USA
[*]Corresponding authors: jonghyeon.ahn@samsung.com, kskim.doc@gmail.com
[+]These authors contributed equally to this work

A Related work

A.1 Manual segmentation

In manual MRI scan segmentation, human raters (e.g., expert physicians) manually delineate and label regions of interest in the scans[1]. Although this method is considered the gold standard, it is cumbersome and has low reproducibility. Manual segmentation of 3D volume scans is generally performed slice-by-slice and typically requires the segmentation of 80 slices, being tedious and time-consuming. While various brain structures have been used to diagnose central nervous system diseases (e.g., stroke, Alzheimer's disease), certain brain structures are used for diagnosing atypical Parkinsonism. The putamen, globus pallidus, midbrain, and pons are the main brain structures that show changes in atypical Parkinsonism and are often segmented for diagnosis and differentiation. However, manual segmentation of these structures in a brain MRI scan is time-consuming and strenuous, even for an experienced radiologist or neurologist who can accurately recognize these structures. In addition, manual segmentation is prone to inter- and intra-rater variability[2–4]. Moreover, the segmentation quality depends on rater proficiency, and even experienced specialists may show variability from their previous annotations. Hence, validation by at least two raters is required for the analysis. Given the challenges and problems of manual segmentation, automated methods are preferred for large-scale datasets in clinical trials or when accurate and quantitative analyses of brain MRI scans are required, such as when measuring the volume or intensity of signals in a brain structure.

A.2 Automated segmentation: Atlas-based method

Automated image segmentation has been dominated by atlas-based methods that formulate segmentation as an image-registration problem[5]. A labeled image (i.e., an atlas) is transformed (i.e., registered) using a deformation model for mapping onto an unlabeled image (i.e., test scan). The established spatial correspondence is then used to transfer labels from the atlas to the target MRI scan[6–8]. Initially, a single atlas delineated by medical experts was used, but segmentation could be highly biased depending on the quality of registration (i.e., the similarity between the atlas and scan)[9]. Subsequently, multiple labeled atlases have been used to mitigate bias and capture wide anatomical variations[9]. Accordingly, two strategies have been proposed: 1) multi-atlas and 2) Bayesian segmentation. Multi-atlas segmentation registers atlases individually onto the test scan and applies label fusion (majority voting) to propagate the most frequently selected labels[10,11]. Bayesian segmentation uses a single probabilistic atlas that summarizes all atlases[12,13]. This entails propagating label probabilities (prior) and image voxel intensities (likelihood) to deduce a generative model (posterior probability) using Bayes' rule. This strategy can be adapted to MRI scans[12,14–16] and is faster than multi-atlas segmentation because it requires only one computationally intensive registration step per scan. Bayesian segmentation is implemented in various tools such as FS[17], statistical parametric mapping[12], and the FMRIB software library (FSL) FMRIB integrated registration and segmentation tool (FIRST)[18].

For our six target brain structures, the average specificity, positive predictive value, and Dice score of FS as higher than those of FSL-FIRST[19]. Additionally, compared with other automated approaches (i.e., statistical parametric mapping and FSL), FS had the highest sensitivity and specificity for brain volume changes in ROC analysis, achieving more consistency, less susceptibility to noise, and better image quality[20–23]. Furthermore, with several segmentation tools introduced for general brain segmentation, FS is frequently used in PD diagnosis[24–27]. Therefore, with the extensive and automated analysis of key features in the human brain, FS has been widely recognized as the most representative atlas-based automated segmentation method for brain structure analysis. Thus, it served as a reference in our study.

A.3 Automated segmentation: DL model

Modern automated image segmentation relies on DL techniques, with the two most generalized DL models being CNNs and ViTs. As for other computer vision tasks, CNNs are predominant in image segmentation owing to the effectiveness of the convolution operation. Convolution deals with sparse interactions (local connections), weight (parameter) sharing, and translation equivariance, giving CNNs a strong and useful inductive bias (prior knowledge) and allowing them to converge quickly with reduced computational complexity. Owing to the effectiveness of the convolution operation, the UNet architecture[28] has achieved outstanding results in the medical field[29–34], being widely used for segmentation. UNet has a U-shaped symmetric encoder–decoder architecture, typically including 1) a convolutional encoder (or downsampling network) to extract relevant features from the inputs at different resolutions, followed by 2) a convolutional decoder (or upsampling network) to synthesize the extracted features as a high-resolution image to obtain pixel- or voxel-wise precision, and 3) a skip connection between layers to recover spatial information lost during downsampling. V-Net[35] is a representative variant of UNet for 3D medical image segmentation.

Despite their efficiency, CNNs have a limited ability to learn long-distance dependencies owing to the locality of receptive fields in the convolutional layers[36,37]. Thus, transformer-based models, which use self-attention mechanisms as core operators, have recently enabled attractive solutions for computer vision tasks. The key idea of the self-attention mechanism, which has shown great success in natural language processing, is to learn the relative importance (self-alignment) of a single token relative to all other tokens in a sequence[38]. In other words, calculating the pairwise interactions between all input units has essentially the same effect as having a global receptive field of long-range dependencies[39]. Inspired by this mechanism, ViT[40] was introduced to interpret an image as a sequence of patches, adapting self-attention for computer vision applications. ViT and its variants have demonstrated excellent performance in many computer vision tasks[41–45]. UNETR[46] is a representative ViT-based 3D image segmentation model that improves the segmentation performance by reducing the loss of encoding information by converting the encoder of an existing CNN-based segmentation model into a ViT. However, transformer-based approaches have limitations, such as the need for large amounts of training data owing to the lack of inductive bias and the quadratic computational complexity of self-attention according to the input image size[40].

Although the medical community is greatly interested in DL models for image segmentation, few studies have been conducted on segmenting intricate brain structures to diagnose diseases. To the best of our knowledge, no existing research has investigated DL methods for segmenting the biomarkers of Parkinsonian syndromes. FS was used as an automated segmentation tool toward diagnosing Parkinsonian syndromes but neglecting DL methods[47,48]. Similarly, a method for fully automated segmentation of the brain without relying on DL was proposed[49]. They introduced a pipeline that uses FS labeling to provide information in a highly nonlinear transformation method (i.e., large deformation diffeomorphic metric mapping). In this study, we used high-performance DL models based on CNN and ViT to segment the brain structures of patients with Parkinsonian syndromes. We established that DL models can yield equal or more effective results than FS. These models can substantially shorten the segmentation time while retaining the accuracy of non-DL FS segmentation.

B Additional qualitative results

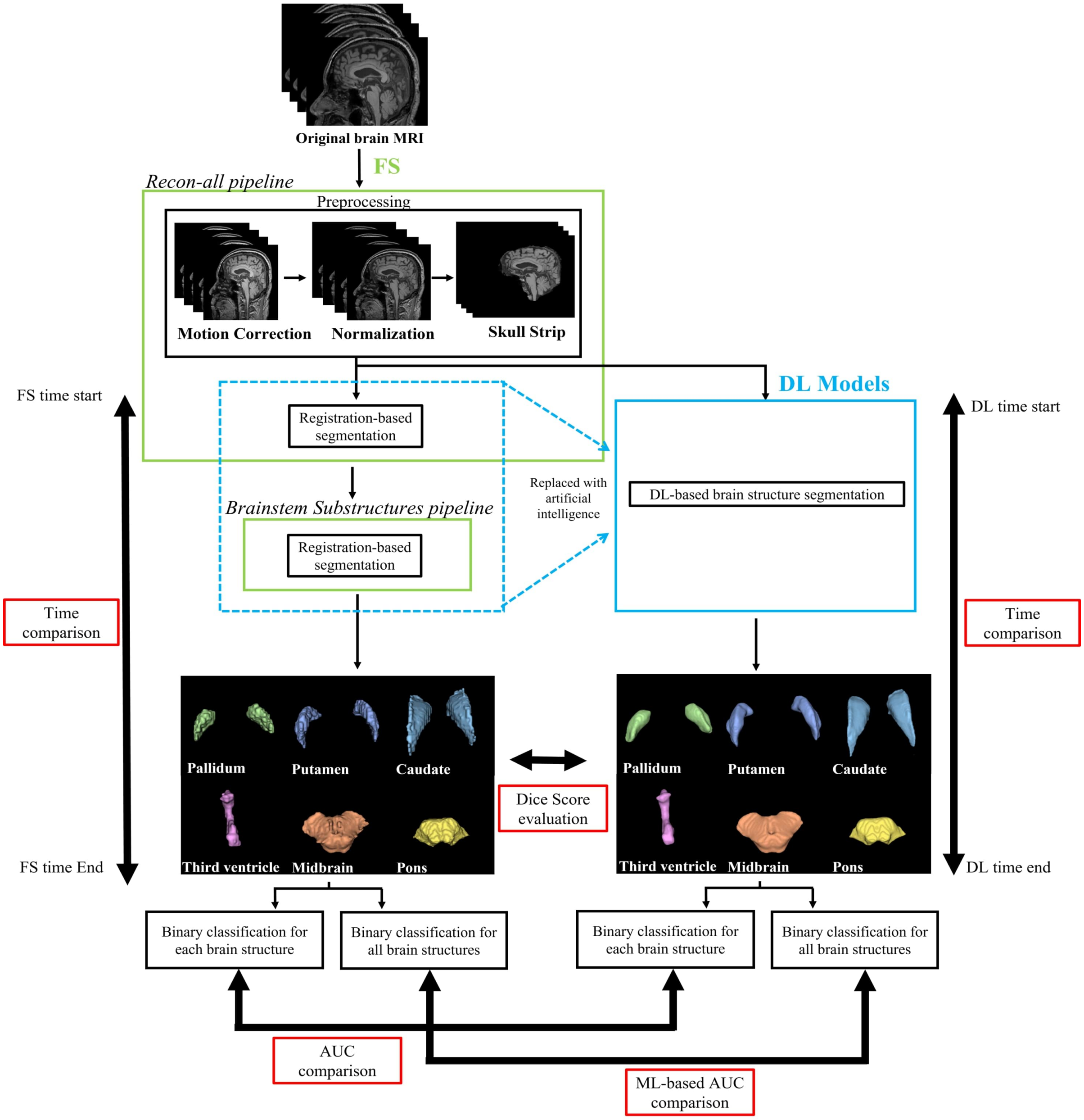

Figure S1. Study overview and performance comparisons. Using FS and DL V-Net and UNETR for segmentation, we analyzed the segmentation time, Dice score, and AUC of disease diagnosis considering each brain structure. In addition, a comparison of AUC was conducted using ML methods for disease diagnosis considering all brain structures.

C Additional quantitative result

	CNN	ViT
Midbrain	0.9385 ± 0.020	0.9642 ± 0.001
Pons	0.9661 ± 0.010	0.9748 ± 0.006
V3	0.9254 ± 0.036	0.9550 ± 0.001
Caudate	0.8892 ± 0.038	0.9456 ± 0.001
Putamen	0.8917 ± 0.028	0.9474 ± 0.002
Pallidum	0.8495 ± 0.045	0.9274 ± 0.002

Table S1. Dice scores of CNN-based V-Net and ViT-based UNETR for brain structures: midbrain, pons, third ventricle (V3), caudate, putamen, and pallidum. The mean $\pm$ standard deviation for threefold cross-validation is provided.

References

1. Zijdenbos, A. P., Dawant, B. M., Margolin, R. A. & Palmer, A. C. Morphometric Analysis of White Matter Lesions in MR Images: Method and Validation. *IEEE Transactions on Medical Imaging* **13**, 716–724, DOI: 10.1109/42.363096 (1994).
2. Massey, L. A. *et al.* Conventional Magnetic Resonance Imaging in Confirmed Progressive Supranuclear Palsy and Multiple System Atrophy. *Movement Disorders* **27**, 1754–1762, DOI: https://doi.org/10.1002/mds.24968 (2012).
3. Schrag, A. *et al.* Differentiation of Atypical Parkinsonian Syndromes with Routine MRI. *Neurology* **54**, 697–697, DOI: 10.1212/WNL.54.3.697 (2000).
4. Kim, Y. E., Kang, S. Y., Ma, H.-I., Ju, Y.-S. & Kim, Y. J. A Visual Rating Scale for the Hummingbird Sign with Adjustable Diagnostic Validity. *Journal of Parkinson's Disease* **5**, 605–612, DOI: 10.3233/JPD-150537 (2015).
5. Pham, D. L., Xu, C. & Prince, J. L. Current Methods in Medical Image Segmentation. *Annual Review of Biomedical Engineering* **2**, 315–337, DOI: 10.1146/annurev.bioeng.2.1.315 (2000).
6. Christensen, G. E., Joshi, S. C. & Miller, M. I. Volumetric Transformation of Brain Anatomy. *IEEE Transactions on Medical Imaging* **16**, 864–877, DOI: 10.1109/42.650882 (1997).
7. Collins, D. L., Holmes, C. J., Peters, T. M. & Evans, A. C. Automatic 3-D Model-Based Neuroanatomical Segmentation. *Human Brain Mapping* **3**, 190–208, DOI: https://doi.org/10.1002/hbm.460030304 (1995).
8. Iosifescu, D. V. *et al.* An Automated Registration Algorithm for Measuring MRI Subcortical Brain Structures. *NeuroImage* **6**, 13–25, DOI: https://doi.org/10.1006/nimg.1997.0274 (1997).
9. Billot, B. *et al.* Automated Segmentation of the Hypothalamus and Associated Subunits in Brain MRI. *NeuroImage* **223**, 117287, DOI: https://doi.org/10.1016/j.neuroimage.2020.117287 (2020).
10. Heckemann, R. A., Hajnal, J. V., Aljabar, P., Rueckert, D. & Hammers, A. Automatic Anatomical Brain MRI Segmentation Combining Label Propagation and Decision Fusion. *NeuroImage* **33**, 115–126, DOI: https://doi.org/10.1016/j.neuroimage.2006.05.061 (2006).
11. Klein, A., Mensh, B., Ghosh, S., Tourville, J. & Hirsch, J. Mindboggle: Automated Brain Labeling with Multiple Atlases. *BMC Medical Imaging* **5**, 1–14, DOI: https://doi.org/10.1186/1471-2342-5-7 (2005).
12. Ashburner, J. & Friston, K. J. Unified Segmentation. *NeuroImage* **26**, 839–851, DOI: https://doi.org/10.1016/j.neuroimage.2005.02.018 (2005).
13. Fischl, B. *et al.* Whole Brain Segmentation: Automated Labeling of Neuroanatomical Structures in the Human Brain. *Neuron* **33**, 341–355, DOI: https://doi.org/10.1016/S0896-6273(02)00569-X (2002).
14. Puonti, O., Iglesias, J. E. & Van Leemput, K. Fast and Sequence-Adaptive Whole-Brain Segmentation Using Parametric Bayesian Modeling. *NeuroImage* **143**, 235–249, DOI: https://doi.org/10.1016/j.neuroimage.2016.09.011 (2016).
15. Van Leemput, K., Maes, F., Vandermeulen, D. & Suetens, P. Automated Model-Based Tissue Classification of MR Images of the Brain. *IEEE Transactions on Medical Imaging* **18**, 897–908, DOI: 10.1109/42.811270 (1999).
16. Wells, W. M., Grimson, W. E. L., Kikinis, R. & Jolesz, F. A. Adaptive Segmentation of MRI Data. *IEEE Transactions on Medical Imaging* **15**, 429–442, DOI: 10.1109/42.511747 (1996).
17. Fischl, B. Freesurfer. *NeuroImage* **62**, 774–781, DOI: https://doi.org/10.1016/j.neuroimage.2012.01.021 (2012).
18. Patenaude, B., Smith, S. M., Kennedy, D. N. & Jenkinson, M. A Bayesian Model of Shape and Appearance for Subcortical Brain Segmentation. *NeuroImage* **56**, 907–922, DOI: https://doi.org/10.1016/j.neuroimage.2011.02.046 (2011).
19. Velasco-Annis, C., Akhondi-Asl, A., Stamm, A. & Warfield, S. K. Reproducibility of Brain MRI Segmentation Algorithms: Empirical Comparison of Local Map PSTAPLE, FreeSurfer, and FSL-first. *Journal of Neuroimaging* **28**, 162–172, DOI: 10.1111/jon.12483 (2017).
20. Dewey, J. *et al.* Reliability and Validity of MRI-based Automated Volumetry Software Relative to Auto-assisted Manual Measurement of Subcortical Structures in HIV-infected Patients from a Multisite Study. *NeuroImage* **51**, 1334–1344, DOI: 10.1016/j.neuroimage.2010.03.033 (2010).
21. Eggert, L. D., Sommer, J., Jansen, A., Kircher, T. & Konrad, C. Accuracy and Reliability of Automated Gray Matter Segmentation Pathways on Real and Simulated Structural Magnetic Resonance Images of the Human Brain. *PLOS ONE* **7**, DOI: 10.1371/journal.pone.0045081 (2012).
22. Mayer, K. N. *et al.* Comparison of Automated Brain Volumetry Methods with Stereology in Children Aged 2 to 3 Years. *Neuroradiology* **58**, 901–910, DOI: 10.1007/s00234-016-1714-x (2016).

23. Klauschen, F., Goldman, A., Barra, V., Meyer-Lindenberg, A. & Lundervold, A. Evaluation of Automated Brain MR Image Segmentation and Volumetry Methods. *Human Brain Mapping* **30**, 1310–1327, DOI: 10.1002/hbm.20599 (2009).

24. Messina, D. *et al.* Patterns of Brain Atrophy in Parkinson's Disease, Progressive Supranuclear Palsy and Multiple System Atrophy. *Parkinsonism @AND@ Related Disorders* **17**, 172–176, DOI: 10.1016/j.parkreldis.2010.12.010 (2011).

25. Vasconcellos, L. F. *et al.* Volumetric Brain Analysis as a Predictor of a Worse Cognitive Outcome in Parkinson's Disease. *Journal of Psychiatric Research* **102**, 254–260, DOI: https://doi.org/10.1016/j.jpsychires.2018.04.016 (2018).

26. Sjöström, H., Granberg, T., Hashim, F., Westman, E. & Svenningsson, P. Automated Brainstem Volumetry Can Aid in the Diagnostics of Parkinsonian Disorders. *Parkinsonism @AND@ Related Disorders* **79**, 18–25, DOI: 10.1016/j.parkreldis.2020.08.004 (2020).

27. Salsone, M. *et al.* Reduced Thalamic Volume in Parkinson Disease with REM Sleep Behavior Disorder: Volumetric Study. *Parkinsonism @AND@ Related Disorders* **20**, 1004–1008, DOI: https://doi.org/10.1016/j.parkreldis.2014.06.012 (2014).

28. Ronneberger, O., Fischer, P. & Brox, T. U-Net: Convolutional Networks for Biomedical Image Segmentation. In Navab, N., Hornegger, J., Wells, W. M. & Frangi, A. F. (eds.) *Medical Image Computing and Computer-Assisted Intervention – MICCAI 2015*, 234–241, DOI: https://doi.org/10.1007/978-3-319-24574-4_28 (Springer International Publishing, 2015).

29. Heinrich, M. P., Oktay, O. & Bouteldja, N. OBELISK-Net: Fewer Layers to Solve 3D Multi-Organ Segmentation with Parse Deformable Convolutions. *Medical Image Analysis* **54**, 1–9, DOI: https://doi.org/10.1016/j.media.2019.02.006 (2019).

30. Kamnitsas, K. *et al.* DeepMedic for Brain Tumor Segmentation. In *Brainlesion: Glioma, Multiple Sclerosis, Stroke and Traumatic Brain Injuries*, 138–149, DOI: https://doi.org/10.1007/978-3-319-55524-9_14 (Springer, 2016).

31. Oktay, O. *et al.* Attention U-Net: Learning Where to Look for the Pancreas, DOI: 10.48550/ARXIV.1804.03999 (2018).

32. Shen, D., Wu, G. & Suk, H.-I. Deep Learning in Medical Image Analysis. *Annual Review of Biomedical Engineering* **19**, 221–248, DOI: 10.1146/annurev-bioeng-071516-044442 (2017).

33. Tong, Q., Ning, M., Si, W., Liao, X. & Qin, J. 3D Deeply-Supervised U-Net Based Whole Heart Segmentation. In *Statistical Atlases and Computational Models of the Heart. ACDC and MMWHS Challenges*, 224–232, DOI: https://doi.org/10.1007/978-3-319-75541-0_24 (Springer, 2017).

34. Zeng, G. *et al.* 3d U-Net with Multi-Level Deep Supervision: Fully Automatic Segmentation of Proximal Femur in 3D MR Images. In *Machine Learning in Medical Imaging*, 274–282, DOI: https://doi.org/10.1007/978-3-319-67389-9_32 (Springer, 2017).

35. Milletari, F., Navab, N. & Ahmadi, S.-A. V-Net: Fully Convolutional Neural Networks for Volumetric Medical Image Segmentation. In *2016 Fourth International Conference on 3D Vision (3DV)*, 565–571, DOI: 10.1109/3DV.2016.79 (2016).

36. Yu, F. & Koltun, V. Multi-scale Context Aggregation by Dilated Convolutions, DOI: 10.48550/ARXIV.1511.07122 (2015).

37. Zhao, H., Shi, J., Qi, X., Wang, X. & Jia, J. Pyramid Scene Parsing Nnetwork. In *Proceedings of the IEEE Conference on Computer Vision and Pattern Recognition*, 2881–2890, DOI: 10.48550/ARXIV.1612.01105 (2017).

38. Bahdanau, D., Cho, K. & Bengio, Y. Neural Machine Translation by Jointly Learning to Align and Translate, DOI: https://doi.org/10.48550/arXiv.1409.0473 (2014).

39. Vaswani, A. *et al.* Attention is All You Need. *Advances in Neural Information Processing Systems* **30** (2017).

40. Dosovitskiy, A. *et al.* An Image is Worth 16x16 Words: Transformers for Image Recognition at Scale, DOI: 10.48550/ARXIV.2010.11929 (2020).

41. Zhu, X. *et al.* Deformable Detr: Deformable Transformers for End-to-End Object Detection, DOI: 10.48550/ARXIV.2010.04159 (2020).

42. Zheng, S. *et al.* Rethinking Semantic Segmentation from a Sequence-to-Sequence Perspective with Transformers. In *Proceedings of the IEEE/CVF Conference on Computer Vision and Pattern Recognition*, 6881–6890, DOI: https://doi.org/10.48550/arXiv.2012.15840 (2021).

43. Kumar, M., Weissenborn, D. & Kalchbrenner, N. Colorization Transformer, DOI: 10.48550/ARXIV.2102.04432 (2021).

44. Chen, H. *et al.* Pre-trained Image Processing Transformer. In *Proceedings of the IEEE/CVF Conference on Computer Vision and Pattern Recognition*, 12299–12310 (2021).

45. Arnab, A. *et al.* Vivit: A Video Vision Transformer. In *Proceedings of the IEEE/CVF Conference on Computer Vision and Pattern Recognition*, 6836–6846 (2021).

46. Hatamizadeh, A. *et al.* Unetr: Transformers for 3D Medical Image Segmentation. In *Proceedings of the IEEE/CVF Conference on Computer Vision and Pattern Recognition*, 574–584 (2022).

47. Bocchetta, M. *et al.* Automated Brainstem Segmentation Detects Differential Involvement in Atypical Parkinsonian Syndromes. *Journal of Movement Disorders* **13**, 39–46, DOI: 10.14802/jmd.19030 (2020).

48. Manjón, J. V. *et al.* pBrain: A Novel Pipeline for Parkinson Related Brain Structure Segmentation. *NeuroImage: Clinical* **25**, 102184, DOI: https://doi.org/10.1016/j.nicl.2020.102184 (2020).

49. Ali R. Khan, M. F. B., Lei Wang. Freesurfer-initiated Fully-automated Subcortical Brain Segmentation in MRI using Large Deformation Diffeomorphic Metric Mapping. *NeuroImage* **41**, 735–746, DOI: https://doi.org/10.1016/j.neuroimage.2008.03.024 (2008).
